# Cytotoxic and Antiproliferative Effect of Tepary Bean Lectins on C33-A, MCF-7, SKNSH, and SW480 Cell Lines

**DOI:** 10.3390/molecules19079610

**Published:** 2014-07-07

**Authors:** Carmen Valadez-Vega, José A. Morales-González, María Teresa Sumaya-Martínez, Luis Delgado-Olivares, Areli Cruz-Castañeda, Mirandeli Bautista, Manuel Sánchez-Gutiérrez, Clara Zuñiga-Pérez

**Affiliations:** 1 Instituto de Ciencias de la Salud. Universidad Autónoma del Estado de Hidalgo. Ex Hacienda la Concepción s/n. Carr. Pachuca-Tilcuautla C.P. 42060 Tilcuautla, Hidalgo, Mexico; E-Mails: ldelgado@uaeh.edu.mx (L.D.-O.); arely_cc@hotmail.com (A.C.-C.); mirandeli@hotmail.com (M.B.); spmtz68@yahoo.com.mx (M.S.-G.); zupecl@yahoo.com.mx (C.Z.-P.); 2 Laboratorio Medicina de Conservación, Escuela Superior de Medicina, Instituto Politécnico Nacional, Plan de San Luis y Díaz Mirón s/n, Unidad Casco de Santo Tomas, México D.F. 11340, Mexico; E-Mail: jmorales101@yahoo.com.mx; 3 Secretary of Research and Graduate Studies, Autonomous University of Nayarit, Ciudad de la Cultura “Amado Nervo”, Boulevard Tepic-Xalisco S/N. Tepic, Nayarit, 63190 Mexico; E-Mail: teresumaya@hotmail.com

**Keywords:** tepary bean, lectins, cytotoxic

## Abstract

For many years, several studies have been employing lectin from vegetables in order to prove its toxic effect on various cell lines. In this work, we analyzed the cytotoxic, antiproliferative, and post-incubatory effect of pure tepary bean lectins on four lines of malignant cells: C33-A; MCF-7; SKNSH, and SW480. The tests were carried out employing MTT and ^3^[H]-thymidine assays. The results showed that after 24 h of lectin exposure, the cells lines showed a dose-dependent cytotoxic effect, the effect being higher on MCF-7, while C33-A showed the highest resistance. Cell proliferation studies showed that the toxic effect induced by lectins is higher even when lectins are removed, and in fact, the inhibition of proliferation continues after 48 h. Due to the use of two techniques to analyze the cytotoxic and antiproliferative effect, differences were observed in the results, which can be explained by the fact that one technique is based on metabolic reactions, while the other is based on the ^3^[H]-thymidine incorporated in DNA by cells under division. These results allow concluding that lectins exert a cytotoxic effect after 24 h of exposure, exhibiting a dose-dependent effect. In some cases, the cytotoxic effect is higher even when the lectins are eliminated, however, in other cases, the cells showed a proliferative effect.

## 1. Introduction

Worldwide cancer-related mortality has increased considerably, thus the need to search for novel drugs and alternative treatments to solve this health problem. One of these alternatives has suggested the use of diverse alternative treatments such as herbal medicines, something that has been known for many decades by humans [1,2,3].

The study of the seeds of legumes and cereals has increased noticeably because these contain a great variety of compounds that display diverse biological activities, such as lectins, which are a very abundant type of proteins in these types of seeds [4,5]. Lectins have been widely studied by many investigators with the purpose of knowing their characteristics, properties, and biological functions [6,7,8,9,10]; however, today one of the studies that researchers are carrying out with lectin is on its capacity for differentiating between normal and malignant cells, which is due to their key characteristic of specifically recognizing carbohydrates, which varies appreciably according to the different ways these proteins are obtained [11,12,13,14]. Lectins have the ability to bind specifically and reversibly to carbohydrates and glycoconjugates, without altering the structure of the glycosyl ligand [15,16,17]. They are found not only in plants, but also in other organisms such as viruses, bacteria, and animals, and they have been shown to possess important biological activities [18,19]. One of the key characteristics of lectins is their capacity to agglutinate cells, particularly the erythrocytes of diverse animals, which is actually employed to identify lectins [7,20,21]. Several lectins are toxic to mammalian cells both *in vitro* and *in vivo*, inhibit growth when incorporated into the diet, and are toxic when injected into animals; therefore, it has been suggested that lectins be utilized as an alternative treatment for some diseases, such as cancer [22,23,24,25].

Cell surfaces, especially mammalian cell surfaces, are heavily coated with complex oligosaccharides, and these glycans have been implicated in many functions, such as cell-to-cell communication, host-pathogen interactions, and cell-matrix interactions [26,27]. Not surprisingly then, glycosylation aberrations are usually indicative of the onset of specific diseases, such as cancer. Therefore, glycans are expected to serve as important biomarkers for disease diagnosis and/or prognosis [28,29,30]. Due to the differences in the sugars of the cellular surface, numerous studies have been conducted to demonstrate that cell-surface carbohydrate-containing molecules are modified after cell transformation. The findings have demonstrated that the expression of cell-surface carbohydrate-binding proteins is also altered by transformation [28,31,32].

It has been observed that alterations on the cell surface are very common in diverse types of cancer, and some of these are well known as progression markers. However, in each cancer type, different patterns of alteration present in the diverse disease stages [33]; thus, changes in glycosylation involve changes in interactions with lectins that can alter the response of cancer cells. Therefore, knowledge of the type and level of lectin interaction with cells and the manner in which they can affect the biology of the tumor will explain the role of carbohydrates in the acquisition of the malignant status, thus its inhibition [11,33,34,35,36].

The study of lectins has led to their use as a tool for the recognition of diverse cell types, such as red blood cells, lymphocytes, sperm, bacterial viruses, and tumor cells. In addition, this has also allowed lectins to be utilized for the study of the processes of transformation of diverse cells, for recognition of the carbohydrates of the cellular membrane, and of the importance of these in diverse cellular processes. One of the uses of lectins of greatest importance in recent decades has suggested that they represent a useful tool for studying the transformation processes of cancerous cells, because it has been observed that lectins exhibit a different agglutination pattern from that of normal cells [37,38], which renders them useful for employment in the detection of malignant changes in transformed cells due to changes on the cancer cell surface [39,40,41]. The anticancer properties of lectin *in vitro* and *in vivo* have been reported by diverse researchers, demonstrating that lectins preferentially hinder T lymphocyte proliferation, and inhibit tumor growth [42,43,44,45]. It has been observed that lectins from different sources, such as plants, inhibit cancer cell growth depending on their concentration and in a differential manner, as well as also depending on the origin of the cells, making it possible to observe very diverse interaction patterns, due to the characteristics of each cell line [34,42,46]. The ability of lectins to modulate growth, differentiation, proliferation, and apoptosis is mainly mediated by surface receptors, such as carbohydrates [47].

Tepary bean (*Phaseolus acutifolius*) lectins have been studied by several researchers to evaluate their cytotoxic effect on normal and malignant cells, from animals and human sources, and it has been observed that in all studied cells, these lectins, whether pure or as protein extracts rich in lectins, showed a cytotoxic effect; in addition, such an action has been associated with glycosylation of the cell surface, as well as with the cell’s nature. In general, a dose-dependent effect has been observed, and in some cases, it has also been observed that cytotoxicity depends on the treatment time. In general, the tepary bean lectin has shown to be able to recognize selectively different types of cancer cells, and between normal and cancer cells [7,48,49,50,51]. However, there have been no reported studies that examine whether the cytotoxic effect of lectins continues when these have been removed from the cell culture medium. Thus, in this paper, we proposed to analyze the cytotoxic, antiproliferative, and post-incubatory effect of the pure tepary bean lectins (TBL) on human malignant cells; C33-A, MCF-7, SKNSH, and SW480 using MTT and ^3^[H]-thymidine assays.

## 2. Results

### 2.1. Tepary Bean Lectin Purification

**Figure 1:**
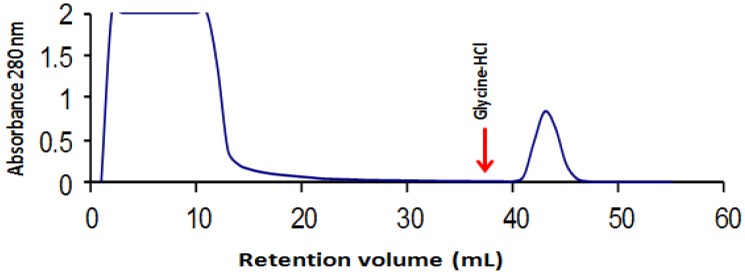
Affinity chromatography in immobilized fetuin of the aqueous extract from tepary beans after precipitation with ammonium sulfate. The lectin fraction was eluted with an acid solution (50 mM glycine-HCl, pH 2.5).

Lectin purification by fetuin-agarose affinity column showed that the lectin was recovered in one peak. The presence of the lectin was detected by a hemagglutination assay, as indicated in previous reports [48], demonstrating that lectin was present in the collected fraction. This purified lectin was employed for biological assays. Figure 1 illustrates the affinity chromatography, in which we can observe that the lectin fraction was recovered when the affinity column was eluted with glycine 50 mM, pH 2.5.

### 2.2. Cytotoxic and Antiproliferative Effect of the Tepary Bean Lectin (TBL)

Initial experiments were conducted to examine the cytotoxic effect of TBL employing the 3-(4,5-dimethylthiazol-2-yl)-2,5 diphenyltetrazolium bromide (MTT) assay. The MTT assay is a colorimetric assay for assessing cell viability, however, tetrazolium dye assays can also be used to measure cytotoxicity (loss of viable cells) or cytostatic activity (shift from proliferative to resting status) of potential medicinal agents and toxic materials.

**Figure 2:**
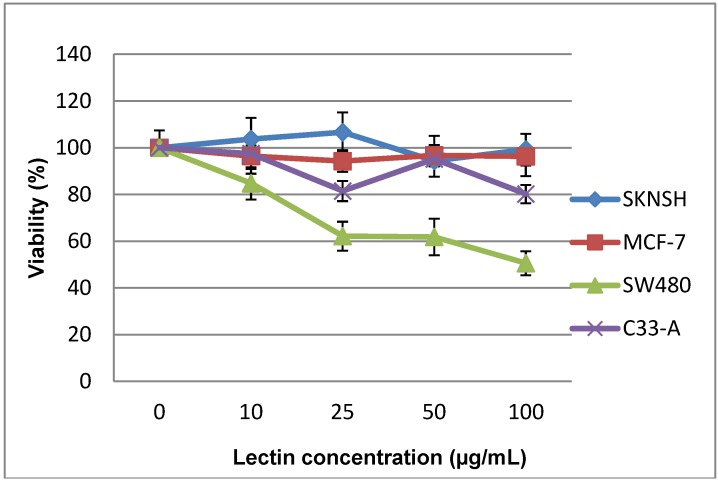
Cytotoxicity of tepary bean lectin (TBL) toward SKNSH, SW480, MCF-7, and C33-A cells after 24 h of exposure employing the 3-(4,5-dimethylthiazol-2-yl)-2,5-diphenyltetrazolium bromide (MTT) cell assay. All values are expressed as mean ± SEM of three independent experiments.

The cytotoxicity of the TBL to SKNSH, SW480, MCF-7, and C33-A cells after 24 h of exposure is depicted in Figure 2. The results of this experiment demonstrated that the tested TBL concentrations did not exhibit significant statistical differences in any of the cell lines. On the other hand, on comparison among cell lines, it was observed that the SW480 cell line was different from the remaining cell lines at all concentrations. In the same figure, it can be observed that SKNSH, MCF-7, and C33-A cells lines did not show significant changes in cell viability with the different lectin concentrations, which indicates that the lectin does not exhibit a cytotoxic effect; however, SW480 cells clearly demonstrated a dose-dependent cytotoxic effect, which indicated decreasing cell viability.

**Figure 3:**
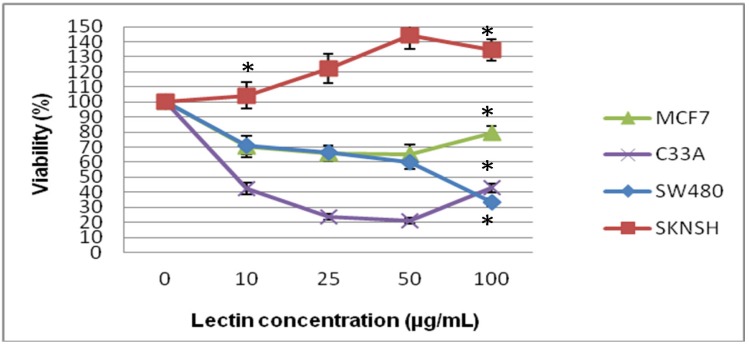
Effect of tepary bean lectin on cell proliferation after 24 h of exposure, in SKNSH, SW480, MCF-7, and C33-A cells employing the ^3^[H]-thymidine assay. All values are expressed as mean ± SEM of three independent experiments.

Studies carried out employing tritium-labeled thymidine (shown in Figure 3) indicated that in SKNSH cells, viability increased when the lectin concentration increased, which indicated that the lectin was not cytotoxic, and SW480 is the cell line in which it can be observed that lectins inhibit cell viability, showing cytotoxic effects. On the other hand, in MCF-7 cells, we can observe a decrease of viability at low lectin concentrations, but when the concentration increased, viability increased, which indicates that a low concentration was cytotoxic, but not at higher concentration. With C33-A cells, a decrease can be clearly observed up to 5 µg/mL, but at 100 µg/mL, the cells exhibited a recovery and viability increased, indicating that cell proliferation is stimulated at higher lectin concentrations. The four cell lines showed significant difference at a dose of 100 µg/mL; however, at the low-dose, a statistically significant difference only presented in SKNSH cells at a dose of 10 µg/mL.

To establish whether the effect of lectin toxicity continues when this substance has been eliminated from the cell culture, we carried out another assay in which, after a 24-h lectin exposure, these proteins were removed and cytotoxicity and proliferation was again determined after 24 and 48 h of incubation with only culture medium, employing MTT and ^3^[H]-thymidine assays.

**Figure 4:**
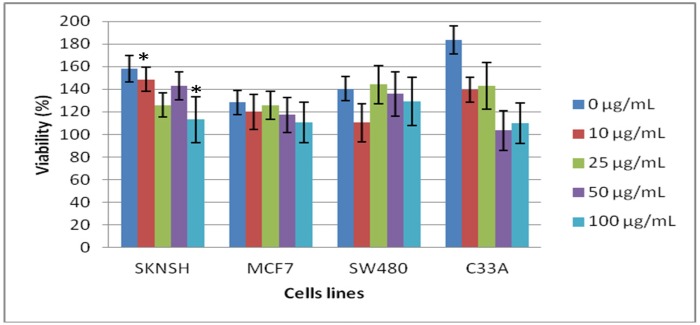
Cytotoxicity of lectin after 24 h, during which lectins were removed from the cells employing the 3-(4,5-dimethylthazol-2-yl)-2,5-diphenyltetrazolium bromide (MTT) cell assay. The cells were previously incubated for 24 h with the lectin solution; then, the solutions were removed and the cells were incubated for 24 h only with cell culture solutions [Dulbecco’s modified Eagle’s medium (DMEM) and fetal bovine serum (FBS)]. All values are expressed as mean ± SEM of three independent experiments.

The results obtained by MTT assay are depicted in Figure 4. These results indicate that after a 24-h incubation, after which lectin was eliminated, all of the cell lines demonstrated diminished cell viability, indicating cytotoxicity and antiproliferative effect of lectins. Statistical analyses of each cell line at the different concentrations of lectins showed that SKNSH cells at lectin concentrations of 10 and 100 µg/mL demonstrated a significant statistical difference, and that the remaining cell lines did not exhibit statistical differences at any lectin concentration. On the other hand, when we compared each lectin concentration among the cell lines, we found that only SW480 demonstrated statistical differences, but that the remaining cell lines did not.

These results indicated that once the lectin was removed from the cells, the cytotoxic effect of these proteins continued, causing the cells to inhibit their proliferation after 24 h of incubation, which at this point indicated that the lectins were attached to the cell membranes. This prevents the cells from being able to metabolize the MTT, which is reflected in the decreased production of the formazan compound. However, at this point, we are unable to know with certainty whether the cells are able to maintain this effect, thereby continuing cell incubation without lectin for an additional 24 h, up to 48 h.

**Figure 5:**
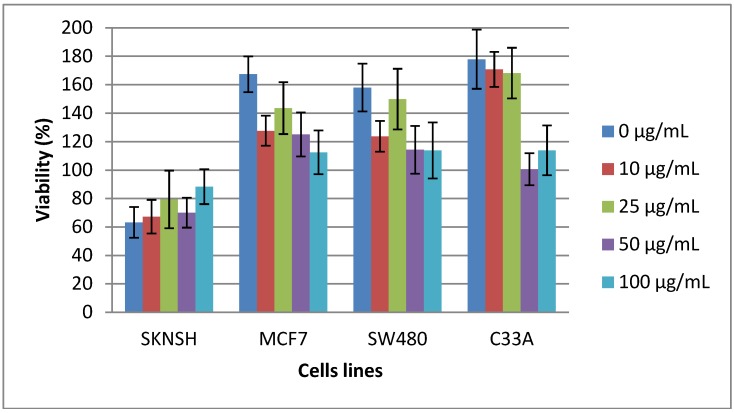
Cytotoxicity of lectin after 48 h, showing that the lectins were removed from the cells, employing the 3-(4,5-dimethylthiazol-2-yl)-2,5-diphenyltetrazolium bromide (MTT) cell assay. The cells were previously incubated for 24 h with the lectin solution. Then, the solution was removed and the cells were incubated for only 48 h with the cell culture solution [Dulbecco’s modified Eagle’s medium (DMEM) and fetal bovine serum (FBS)]. All values are expressed as mean ± SEM of three independent experiments.

When the cell proliferations were analyzed after 48 h of incubation without lectin, the results illustrated in Figure 5 indicated that in the SKNSH cell line, viability increased when the lectin concentration increased; in MCF7, SW480, and C33-A cell lines, we found that cell viability decreased when the lectin concentration increased. However, in statistical terms, no cell lines showed significant differences, nor did we observe statistical differences among cell lines at different lectin concentrations.

As can be observed, some of the cell lines continued to decrease in viability; SKNSH cells, however, were able to recover and increase their cell viability. These results may indicate that although the lectin caused damage, these cells are able to repair this damage and to continue to perform certain metabolic functions, or that the level of damage caused by the lectins was not permanent and that cell proliferation could be recovered at some time after the lectins were removed from the culture medium. However, on the other hand, in the remaining cell lines, damage was more severe, perhaps because the lectins were more strongly bound to the cells and were unable to recover.

**Figure 6:**
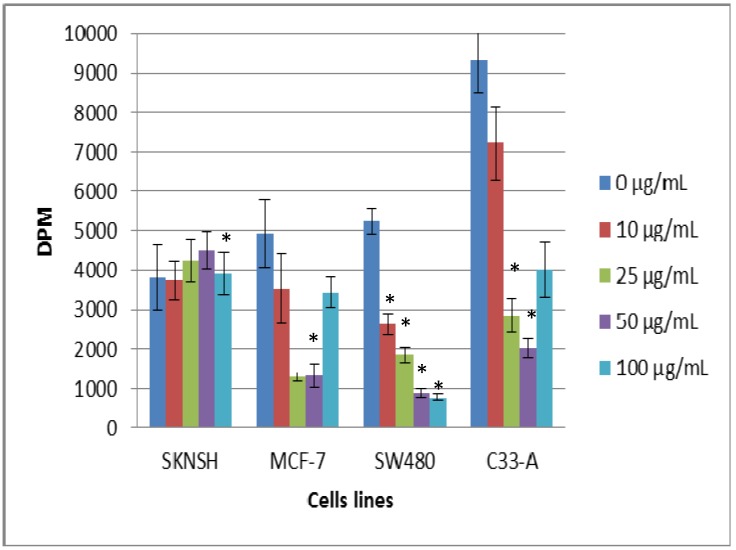
Effect of lectin on cell proliferation after 24 h during which lectins were removed from the cells, employing the ^3^[H]-thymidine assay. The cells were previously incubated for 24 h with lectin solution; then, the solution was removed and the cells were incubated for only 24 h with the cell culture solutions [Dulbecco’s modified Eagle’s medium (DMEM) and fetal bovine serum (FBS)]. All values are expressed as mean ± SEM of three independent experiments.

The post-incubatory assay carried out employing is shown in Figure 6. We can observe that after 24 h of incubation without lectins, SKNSH cells increased their cell proliferation from 0 to 50 µg/mL, but at 100 µg/mL, proliferation decreased, being statistically significant this reduction. MCF-7 cells exhibited a significant decrease in proliferation, however, at the higher concentration, the cells were capable of recovery and increased proliferation, being only statistically different at 50 µg/mL. SW480 was the only cell line that showed a continuous decrease in proliferation at all of the lectin concentrations analyzed, this decrease being statistically significant, while on the other hand, C33-A demonstrated a decrease in cell proliferation; this reduction to concentrations of 25 and 50 µg/mL was statistically significant but, similarly to that of MCF-7, cell proliferation increased at 100 µg/mL.

**Figure 7:**
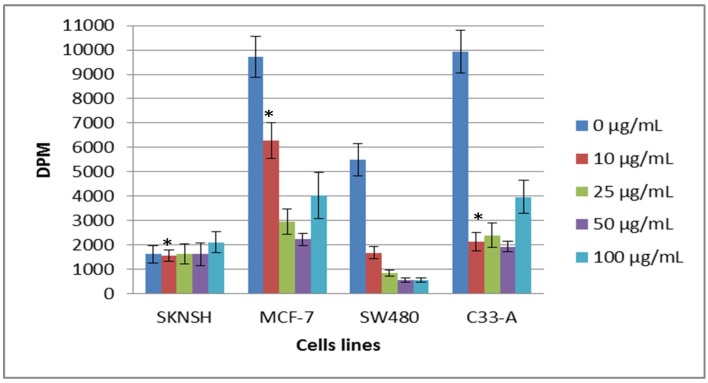
Effect of lectin on cell proliferation 48 h after the lectins were removed from the cells, employing the ^3^[H]-thymidine assay. Cells were previously incubated for 24 h with the lectin solutions; then, the solutions were removed and the cells were incubated for only 48 h with the culture cell solutions [Dulbecco’s modified Eagle’s medium (DMEM) and fetal bovine serum (FBS)]. All values are expressed as mean ± SEM of three independent experiments.

The results when proliferation was measured after 48 h without lectins are illustrated in Figure 7. SKNSH cells showed a considerable increase of proliferation in all concentrations tested, and it was only significant at 10 µg/mL. MCF-7 cells showed a decrease in proliferation up to 50 µg/mL, but 100 µg/mL presented a significant increase; however, only at 10 µg/mL were statistically significant differences found. In SW480 cells, proliferation continued to decrease at all lectin concentrations analyzed, but without a statistical difference, and in C33-A cells, proliferation decreased, but an increase was observed at 100 µg/mL.

These results indicated that at low concentrations, the cytotoxic effect of lectins was maintained, even if these were not still present in the culture medium. However, in lectins that is bound strongly the damage continue although the lectin solution was retired, but at higher concentrations of 100 µg/mL, recovery cells exhibited activity, thus an increase in the ability to divide by increasing the amount of thymidine incorporated into the DNA.

## 3. Discussion

Many studies have reported the purification of the lectins of several vegetable sources, including ion exchange and affinity [48,52,53,54]. In previous articles, our group reported the purification of the lectin of the white tepary bean employing fetuin agarose as array, obtaining good results in purification [48], as indicated in this article. Likewise, we have previously reported that lectin possesses high affinity to type A human erythrocytes, which has indicated to us that the lectin purified using this methodology offers high biological activity, which is an indicator that the purified protein corresponds to the lectin.

During lectin purification by affinity chromatography, it was determined that the fraction not retained by the matrix continued to demonstrate considerably high agglutination activity. This is probably due to that the fetuin matrix does not possess the ability to retain some of the lectin isoforms contained in the beans, retaining in this manner only a fraction of these. There are reports that indicate that lectins of different bean varieties consist of several isoforms and that in order to purify these independently, it is necessary to employ certain chromatography techniques, either independently or in combination, such as ion exchange or affinity, because each isoform possesses a different purification pattern [55,56,57].

When we analyzed the pure lectin by sodium dodecyl sulfate-polyacrylamide gel electrophoresis (SDS-PAGE) under reducing conditions (data not shown), this exhibited only one band with a molecular weight of approximately 31 KDa, which led us to think that only one protein was purified. However, previously published studies in which this same affinity matrix was employed showed that such a matrix not only retains a single isoform, but also receives a mixture of all three of these, which differ little in their isoelectric points and molecular weights [48]. Another report indicated that lectin isoforms of the tepary bean can be purified using ion exchange chromatography, affinity chromatography, and electrofocusing chromatography. In addition, various chromatographic techniques have been reported for the purification of lectins from other vegetables, such as ion-exchange and affinity chromatography, in order to be able to separate the lectin isoforms properly [56,58,59].

The MTT technique is known as an assay that is based on the ability of living cells to transform 3-(4,5-dimethylthiazol-2-yl)-2,5-diphenyltetrazolium bromide (MTT) by means of the action of the mitochondria into a colorful formazan product, which is a direct indicator of the amount of viable cells, and also can be used to measure cytotoxicity or cytostatic activity (shift from proliferative to resting status); however, there was a degree of error, due to that there may be cells that do not exhibit immediate metabolic activity or even that their activity is decreased due to the action of the lectin bonding to the cells. Although this methodology is well accepted for carrying out feasibility and cell proliferation studies, there are other methodologies that can yield better results concerning the cells’ biological activity, such as the technique that includes DNA synthesis, as well as the use of tritiated thymidine.

The results observed in the study with the MTT assay indicate that there is great variability in the cytotoxic effect of lectins on the four cell lines studied. It is noteworthy that these cell lines derive from different cancer origins, but it has been documented that there are significant changes in the pattern of the glycosylation of cell membranes in the carcinogenic process. The binding of lectins to cell membranes depends on the type of carbohydrate to which the lectin is related, and also to that found in the cell membrane and, on the other hand, to the strength of the binding of these lectins. As we can observe, although in all cell lines the lectins were most likely bound by the union pattern, this was considerably variable and the quantity of lectins bound was also different, all of this due to the differences of each cell line, as well as to that the damage caused by the lectin in each cell line is varied, despite that statistically, not a great difference has been noted among the lines. As mentioned previously, the cell line that exhibited the greatest effect was SW480; this could have been due to that lectin bound in a greater quantity and exerted a greater toxic effect on these cells, in contrast with the remaining cell lines studied.

With the use of the tritiated thymidine technique, we are very well aware that this is a much more accurate technique than the MTT one for directly measuring the amount of thymidine incorporated by living cells and their capability of dividing, which renders it a better technique for measuring lectin-associated cellular damage. Thus, this technique more clearly shows the level of damage caused by the lectins in each of the cell lines.

As already reported in a previous work, pure TBL, as well as other varieties of beans and vegetables, have demonstrated a cytotoxic effect on various human malignant cell lines, and also an animal cytotoxic effect [7,49,50], as well as *in vivo*, causing damage to several cells in organs [22].

In this work, we have shown that TBL possess a significant cytotoxic effect on the studied cell lines, and that this cytotoxic effect is dose-dependent. On the other hand, it was noteworthy that regardless of the technique employed, SW480, C33-A, and MCF-7 cell lines demonstrated inhibition in cell proliferation, while the SKNSH line showed a slight increase in cell proliferation when the lectin concentration was increased.

Some studies have reported that when malignant cells are exposed to different concentrations of lectin or lectin-containing protein extracts, these cells undergo severe damage, greatly decreasing cell viability and cell proliferation, and that this damage increases with increased lectin concentration and increased exposure time of the cells [7,48,49,50]. However, little is known about the capacity or the lack of capacity of cell recovery once the lectin solution is removed from the cell culture. Thus, in this work, we performed the task of studying the recovery ability of the malignant cell, and it was shown that cell recovery was dose-dependent and that the former also depended on the nature of the cells studied.

Cells such as SW480, C33-A, and MCF-7 showed that although the lectin solution was withdrawn 24 or 48 h prior to measurement of cell proliferation, the damage that this lectin caused to the cells when they were exposed for 24 h was sufficient to avoid cell proliferation at low concentrations. This can be explained by the nature of lectins, due to the affinity that lectins possess toward certain types of carbohydrates and, on the other hand, to the characteristics of the cells themselves. It is well known that each normal or malignant cell line has different types of carbohydrates on the outside of the cell membrane, allowing or not the binding of certain lectins; thus, the lectin of the tepary beans studied in this work exhibited different patterns of affinity to the carbohydrates present in the cells under study.

In the case of the SKNSH cell line, the results observed in this work are different from those of the remaining three cell lines. When the cells were initially exposed to the lectin, they displayed a marked reduction in cell proliferation; however, when the lectin was withdrawn, the cells exhibited recovery. This event can be explained due to the binding between the lectin and the carbohydrates present in the cells, because even though the cells are exposed to the lectin, these can cause cellular damage sufficient to reduce proliferation. On the other hand, when the lectins are removed from the culture medium due to that lectins are not strongly attached to the cells, the lectins can be easily eliminated during cell washings, thus not allowing the lectins to continue causing damage to the cells. Therefore, it is has been shown that in the absence of lectins, cells have the capacity for recovery, which is reflected in the increase in cell proliferation.

One important observation is that in the four cell lines, the greatest recovery of cells was observed at the highest concentration evaluated (100 µg/mL); such a recovery at that concentration can be explained as indicated by Stanley and Carver [60], who reported that in some cells, on being exposed to high concentrations of lectins, cell mutation is promoted, producing lectin-resistant cells with the ability to proliferate in the presence of lectins.

Comparing the post-incubation test with respect to time, important differences were observed in the results of the two techniques employed, which was to be expected; therefore, as mentioned previously, these two techniques have vastly different fundamentals. However, in a general manner, after 24 h of post-incubation, it was noted that cell lines SKNSH, SW480, and C33-A exhibited a trend toward continuing the inhibition of proliferation, indicating a sustained effect of the lectin. On the contrary, MCF-7 cells possessed the capacity to recover over time, an increased cell proliferation was observed. These results show the differences that exist in lectin-carbohydrate interactions in the different cell lines, and given that the selectivity of the lectins is very important for interaction or not, with this study we can make a selection among the four cell lines, thus proposing which lines would be the most appropriate to employ to continue with more specific studies on the specificity of carbohydrates and mechanisms of action, inhibition, among others.

From a clinical point of view, for the use of lectins, it is important to find cells, glycoconjugates, or carbohydrates to which these present high specificity; therefore, in the case of tepary lectins, it is of great interest to identify these, because in this manner these proteins will possess much greater potential for use for clinical purposes. This is the case of some other vegetable lectins, which have already been widely studied and which today are used for clinical purposes, such as in histopathology, immunology, glycoprotein characterization, transformation, and cell inhibition [61,62,63].

It has been reported that when lectins are in contact with cells, they can bind with carbohydrates on the cell surface, or they can be internalized into the cells and can trigger a wide variety of signals, such as induction of apoptosis or cell cycle arrest, downregulation of telomerase activity, and inhibition of angiogenesis, activating the immune system by stimulating the proliferation of T lymphocytes, ribosomal inactivation, increasing tumor necrosis factor alpha (TNF)-α and inhibiting the release of anti-inflammatory interleukin (IL)-10 [4,5,42,47,64,65,66,67,68].

What has been reported up to now on the cytotoxic effectw of tepary lectins leads us to propose that their cytotoxicity is triggered by the lectins that bind to specific carbohydrates found in the cell membrane, and that this binding is capable of triggering events inside the cell. However, this continues subject to proof, and other possibilities must be considered as well, such as internalization of the lectin. Certainly, at present we do not know the mechanism by which the tepary lectins cause damage to cells, but it is possible that any one of the previously mentioned mechanisms is responsible for the cytotoxic effect. Thus, further studies are needed to elucidate the mechanism.

## 4. Experimental Section

### 4.1. Extraction and Purification of Lectins

Tepary bean (*Phaseolus acutifolius*) seeds were purchased at a local market in Hermosillo (Sonora State, Mexico). The beans were ground in a Wiley mill fitted with a 60-gauge mesh. Tepary lectin was extracted and purified as indicated previously [48]. Briefly, proteins were extracted from the flour after 16 h at 4°C with 10 mM phosphate buffered saline (PBS) solution and 1:10 (w/v) flour:buffer ratio. The lectin was purified by affinity chromatography in a fast protein liquid chromatography (FPLC) system (Amersham Biosciences, Upssala, Sweden) fitted with a fetuin-agarose column, and the bound fraction was eluted from the column with 20 volumes of 50 mM glycine-HCl, pH 2.5.

### 4.2. Cell Culture

The cytotoxicity assay was conducted using the following four human malignant cells lines: MCF-7 (human breast adenocarcinoma); SKNSH (human bone marrow neuroblastoma); SW480 (human Caucasian colon adenocarcinoma), and C33-A (human epithelial cervical carcinoma), which were obtained from the American Type Culture Collection (ATCC, Rockville, MD, USA). The cells were grown in Dulbecco’s modified Eagle’s medium (DMEM, Gibco, Grand Island, NY, USA) with 10% Fetal bovine serum (FBS) (Gibco) in a CO_2_ water-jacketed incubator (Nuaire,Plymouth, MN, USA) at 37 °C in a humidified atmosphere of 5% CO_2_ and 95% air.

### 4.3. Cytotoxic and Antiproliferative Assay

#### 4.3.1. MTT Assay

The tetrazolium dye colorimetric test (3-(4,5-dimethylthiazol-2-yl)-2-5-diphenyltetrazolium bromide (MTT) was used to determine the viability of the cells lines. The MTT assay is based on the ability of functional mitochondria to catalyze the reduction of MTT into insoluble formazan, the concentration of which can be measured spectrophotometrically [69]. SKNSH, SW480, MCF-7, and C33-A cell lines were first cultured in 96 well microplates (5.0 × 10^3^ cells/well) in DMEM for 24 h, washed twice using PBS, and pretreated with different concentrations of TBL (0.10, 25, 50, and 100 µg/mL). After a 24 h incubation, the cells’ lectin solution was removed and we added DMEM supplemented with FBS; incubation was continued for 24 and 48 h. Subsequently, at 24 and 48 h, respectively, the culture medium was eliminated and the cells were washed with PBS, we added MTT reagent (5 mg/mL) to each well, and the plate was incubated at 37 °C for an additional 3 h. The media were then removed and the intracellular formazan product was dissolved in dimethyl sulfoxide (DMSO). The absorbency of each well was then measured at 540 nm and the percentage of viability was calculated. All determinations were carried out in triplicate.

4.3.2. ^3^[H]-Thymidine Assay

The cells were cultured in 24-well flat-bottomed plates at a concentration of 5 × 10^4^ cells/well and incubated at different TBL concentrations (0, 10, 25, 50, and 100 µg/mL) in DMEM for 24 h. After a 24 h exposure to the lectin, the solution was removed, and DMEM supplemented with FBS was added. Incubation was continued for 24 and 48 h and cell proliferation was measured at each period by detection of the incorporation of tritium-labeled thymidine (3 µCi/well) during a 30 min incubation period. To stop the uptake of radioactivity, the medium was removed and the cells were washed twice with phosphate buffer pH 7.6 containing 150 mM NaCl; then, we added 500 µL of SDS 0.1% containing 10 mM ethylenediaminetetraacetic acid (EDTA, pH 7.4) to each well. After 30 min at room temperature, the lysate was added with 500 µL of 10% cold trichloroacetic acid (TCA) and the precipitate was collected on a nitrocellulose paper, washed three times with TCA 5%, dried, and counted in a liquid scintillation counter (Beckman LS 65000, Brea, CA, USA). All determinations were performed in triplicate.

### 4.4. Statistical Analysis

Experimental results are expressed as mean ± SEM. All measurements were replicated three times. The data were analyzed by an analysis of variance (*p* < 0.05), followed by the Tukey post hoc test for multiple comparisons. IC50 values were calculated from linear regression analysis.

## 5. Conclusions

Purification of lectins from tepary beans using the fetuin affinity column was a good choice for purification, however, purification could be improved by employing another affinity matrix with higher affinity for tepary lectins, or some combination with other chromatographic techniques, to improve lectin purification and separate the lectin isoforms. We observed a marked effect of the lectins on the inhibition of cell proliferation in the SW480 cell line, with the effect not being so marked in the remaining three cell lines studied.

The post-incubation results obtained 24 and 48 h after the lectin was eliminated from the cell culture showed that by the MTT technique, the four cell lines studied possessed some recovery capacity, displayed by increasing cell proliferation. On the other hand, post-incubation proliferation results obtained by tritium-labeled thymidine after 24 and 48 h prior to the lectin solution being eliminated from the cell culture showed that SW480 cells were not able to recover their proliferation activity: the C33-A and MCF-7 cell lines did not present recovery at low lectin concentrations, while at high concentrations, both cell lines presented recovery. In the case of SKNSH cells, these demonstrated proliferation activity after elimination of the lectin. Recovery in cell proliferation 24 and 48 h after the lectin was eliminated, as observed in SKNSH, C33-A, and MCF-7 cell lines, was highest at the 100-µg/mL concentration.
